# Research on the Influence of Dynamic Work Environment on Employees’ Innovative Performance in the Post-epidemic Era – The Role of Job Crafting and Voice Behavior

**DOI:** 10.3389/fpsyg.2021.795218

**Published:** 2021-12-14

**Authors:** Jianhua Wang

**Affiliations:** ^1^Evergrande School of Management, Wuhan University of Science and Technology, Wuhan, China; ^2^Business School, Changshu Institute of Technology, Suzhou, China

**Keywords:** dynamic work environment, innovative performance, job crafting, voice behavior, mediation effect

## Abstract

In today’s interconnected world, environmental uncertainty is higher than ever. Under the new economic normal, innovation-driven has become the key to the transformation and upgrading of various enterprises. Employees’ behavior affects the company’s innovative performance, but it is also deeply affected by the dynamic work environment. The sudden epidemic has greatly increased the environmental dynamics and uncertainties faced by individuals, and also caused many changes in individual behavior. However, the research on the mediating mechanism and boundary conditions of how the dynamic work environment affects employee behavior and results is relatively few. Based on uncertainty reduction theory and innovative performance theory, and following the research paradigm of “environment-behavior-performance,” a moderated mediation model with job crafting as the mediating variable and voice behavior as the moderating variable is constructed. Through the statistical analysis of 210 valid questionnaires for employees in different types of enterprises, the mechanism of how the dynamic work environment affects innovative performance by promoting employees to carry out job crafting is discussed. According to the test results, the dynamic work environment has a significant positive impact on individual innovative performance, and job crafting plays a mediating role in the relationship between the two. In addition, voice behavior positively moderate the relationship between dynamic work environment and job crafting, and the indirect relationship between dynamic work environment and innovative performance through job crafting.

## Introduction

With the transformation of enterprises from an industrial economy to a knowledge economy, competition among enterprises has become increasingly fierce. Due to the development of emerging technologies such as “Internet +,” cloud computing, and block-chain, various new industries, new formats, and new models are constantly emerging, bringing huge opportunities and challenges to enterprises. In today’s interconnected world, the environment is more uncertain and dynamic than ever before. Facing the uncertainty and dynamics of the environment, enterprises must solve the problem of how to promote the long-term development of the organization through innovation, so as to improve organizational performance, and maintain a unique competitive advantage in the era of technological iteration and change. Therefore, innovation is not only a trend in increasingly competitive dynamic environment, but also a necessary way for enterprises to gain competitive advantage. The innovative performance of an enterprise depends on the innovation behavior of employees. Employee innovation is the foundation of enterprise innovation. Under the catalysis of time, a certain number of individual-level innovation will bring qualitative changes to the organization and give the organization a new lease of life. Therefore, the continuous accumulation of employee innovation has contributed to the production of organizational innovation, which is one of the decisive factors of organizational innovative performance (Amabile, 1993; Welbourne et al., 1998). In this situation, research on employees’ innovative performance is particularly important. Seeking to stimulate employee innovation and improve the effectiveness of their innovation activities has also aroused widespread concern from academia and enterprises.

Global competition continues to intensify, technology continues to update, and the pressure to survive has become more obvious. These changes have made every organization and individual in a dynamic environment with full of uncertainties (Chen et al., 2018). How to face the dynamic environment with full of challenges and opportunities has become one of the biggest challenges for organizations and individuals. In particular, the sudden outbreak of COVID-19 epidemic has involved many organizations in a huge crisis, causing both organizations and individuals to face a high degree of uncertainty and complexity. The epidemic is a heavy test for everyone, every company, and the entire country and the world. After this epidemic crisis, it must be an accelerated process of elimination and upgrade for both organizations and individuals. Those organizations and individuals with rapid upgrade capabilities will be able to adapt and make full use of the challenges and opportunities brought by the dynamic environment, thereby improving the organization and individual performance. As an effective management measure to deal with complex and dynamic environments, work design has become an important focus of organizational management (Brenninkmeijer and Hekkert-Koning, 2015). Just as under this epidemic, many domestic organizations have chosen the “new work” model of working at home. Different from the traditional office mode, working at home is a working mode that requires online, independent, and collaborative work, which requires work redesign. In traditional management, work design is a top-down process, that is, managers are responsible for restructuring the work of employees. However, one of the criticisms of this approach is that continuous changes and increasing complexity in the work environment are not taken into consideration (Grant and Parker, 2009). Therefore, Grant and Parker (2009) suggested that managers in organizations no longer need to design fixed and static jobs, but need to provide more flexible jobs. In these jobs, employees can actively change and develop tasks on their own to respond to job needs and opportunities.

Effectively stimulating the initiative and creativity of employees in a dynamic and complex environment is the key to a company’s control of uncertainty. The dynamic changes of the environment and the enhancement of employees’ individual awareness mean that organizations need to shift their perspective to the proactive behavior of employees. In the proposition of work design, employees can also actively participate in it, and the concept of job crafting came into being. During the COVID-19 epidemic, medical staff rushed into battle, willing to contribute, and reshaped the mission and value of their job. During the resumption of work, employees worked remotely and reinterpreted their working relationships. The post-90s and even post-00s have embodied the spirit of responsibility of the new generation of employees. Studies have shown that when employees are in an environment with greater work pressure and higher autonomy, they will proactively implement their job crafting (Tims et al., 2013). The job crafting behavior of individuals to work will further affect employees’ performance (Tims et al., 2015; Rudolph et al., 2017). In addition, recent research also pointed out that in increasingly complex and changeable dynamic work environment, employees’ voice behaviors will also play a direct and indirect moderating role.

On the one hand, dynamic work environment will expose employees to a large number of potential development opportunities. On the other hand, dynamic work environment will also bring unforeseen accidents or disturbances to employees’ work and career development. The work pressure faced by employees has increased significantly, and small work errors may cause greater losses (Waldman et al., 2001). In this uncertain situation where challenges and opportunities for change coexist, employees must find ways to adapt to environmental changes, develop and utilize environmental opportunities, and avoid environmental risks. Employees may take the initiative and spontaneously adjust their own status, make more work input, and design and adjust their work content and boundaries. According to resource protection theory and social exchange theory, in the process of job crafting, employees will actively make voice behavior for the purpose of protecting their own resources or creating value for the organization. If employees’ voice behavior is recognized by their bosses and colleagues, employees will be more proactive in finding a match between the individual and the work, and will be more proactive in making voice behavior to the work. Existing research has shown that employees’ voice behavior has a positive effect on the improvement of innovation performance (Ng and Feldman, 2012). With the reduction of social resources, active work input, negative mentality and psychological pressure, employees continue to update their knowledge and improve their work skills at work. All of these provide a strong basic guarantee for employees’ innovation activities and are conducive to the improvement of employees’ innovation performance.

The contribution of this research is mainly reflected in the following aspects: First, it enriches the research between dynamic work environment and its result variables. This research confirms that the cognition of the dynamic work environment can promote the innovation performance of employees, thus enriching and expanding the research between the dynamic work environment and its results. Second, by introducing the mediating variable of job crafting, this research can discover the key role played by the environment characteristic variables of employees, and also supports the important role of job crafting. This provides a new perspective for explaining the relationship between the dynamic work environment and innovation performance, opens the “black box” of positive effects in the dynamic work environment, and provides a good realistic explanation for the performance dilemma of employees in the post-epidemic era. Finally, by constructing a moderated mediation model, it more comprehensively reflects the complex process of the dynamic work environment affecting innovation performance of employee. On the basis of self-loss theory (Prem et al., 2016) and social information processing theory (Frazier and Bowler, 2015), the introduction of voice behavior as a moderating variable confirms the important role of employees’ participation in organizational management.

The following section presents the relevant literature review and forms hypotheses for testing. The methodology of testing the hypotheses is outlined, followed by the analysis of the results. Discussion, implications, conclusion as well as the limitations and future research are provided in the final section.

## Literature Review and Hypotheses

### Dynamic Work Environment and Innovative Performance

Dynamic is a key feature of the environment, which refers to a certain degree of rapid, unpredictable and turbulent changes (Eisenhardt and Martin, 2000). The dynamic environment mainly emphasizes the speed, complexity and way of environmental change (Rodriguez-Pea, 2021), and the uncertainty and unpredictability that it brings (Rosenbusch et al., 2013). Scholars generally divide environmental dynamics into technological development dynamics and market demand dynamics, which mainly include unpredictability of customers, market trend change rates, peer competitors, business growth opportunities, R&D process (Rodriguez-Pea, 2021). In addition, environmental dynamics are related to uncertainty, which weakens the employer’s ability to predict events and their impact on the organization (Lumpkin and Dess, 2001). When the environment is full of dynamics, meeting the latest requirements and changes in the environment may greatly affect the survival and development of the organization. Therefore, how to deal with the uncertainty brought about by the dynamic environment has become an important factor that must be considered in modern organization management (Hmieleski and Ensley, 2007; Ember, 2013; Diakanastasi et al., 2018; Smithson et al., 2019).

The impact of the dynamic work environment on the organization and employees should not be underestimated. The strength of uncertainty affects the behavior of organizational managers and employees in facing environmental changes with the complexity, ambiguity and turbulence (Fiedler, 2015). In a dynamic work environment, both organizations and employees must find ways to deal with the opportunities and challenges that the environment brings. For example, the outbreak of the epidemic has caused many organizations and employees to change their traditional working methods and switch to telecommuting to start and resume work. This dynamic change in the work environment profoundly affects the behavior of individuals in the organization. Studies have pointed out that the adjustment and change of work methods during the epidemic will not only affect the efficiency of the organization and the cost of personnel communication (Glac and Zdebska, 2020), but also have a corresponding impact on the psychology and behavior of employees in the organization (Julio et al., 2020), easy to intensify work-family conflict (Zhou et al., 2020), and even affect employees’ perception of career prospects (Mao et al., 2020). Baron and Tang (2011) pointed out that the dynamics of the environment may be a catalyst for the innovative behavior of organizations and individuals. When faced with uncertainty, people tend to show certain behaviors to reduce uncertainty (Deng et al., 2019). From the perspective of individual attention, employees in a dynamic work environment will pay more attention to changes in the environment. When employees pay more attention to the environment rather than themselves, they will take a more accurate self-assessment in consideration of the overall situation (Aron et al., 1992) and adopt corresponding behavioral strategies to respond positively to uncertain environments, for example, employees do the job crafting to improve work performance.

Employees’ innovative performance is a good idea or result generated and realized by an individual, which can benefit organizational performance (Fan et al., 2016). Employees’ innovative performance is achieved through their innovative activities. Innovative performance includes not only the results of innovation, but also the generation and promotion of innovative ideas and innovation support to the organization (Hagedoorn and Cloodt, 2003). As the output of corporate development strategy, employee innovation performance is an infinite source of sustainable development for an organization (Chen et al., 2016). At the same time, with the profound and complex changes in the environment, in order to seek greater development, organizations have higher and higher requirements and expectations for the innovation ability and innovation performance of the workforce. Previous discussions on the relationship between environmental uncertainty and performance have mostly focused on research at the enterprise or management team level (Li and Zhao, 2016; Deng et al., 2019). For example, in the face of an uncertain environment, organizations can implement mergers and acquisitions, design, and flexible human resource management strategies (Wan and Yiu, 2009), improvisation and reallocation of resources (Tseng et al., 2015), perceive and grasp potential opportunities (Li and Atuahene-Gima, 2001) and other ways to gain a competitive advantage and improve organizational performance. However, dynamics is not only a challenge for organizations and leaders, but also for employees. Küller et al. (2006) believe that the work environment has an impact on human psychology. Especially under this epidemic, many employees have found that their work environment is full of changes, challenges and opportunities. Thomas and Obal (2018) believes that in the case of rapid changes in the external environment, employees’ role pressure will increase, and employees need a wealth of knowledge to balance the negative effects of uncertainty. First, in a dynamic work environment, employees will be more likely to adopt positive behavior strategies to reduce uncertainty, so as to carry out their work more effectively and improve their innovative performance. Second, there are opportunities for change in a dynamic work environment. When individuals can effectively identify opportunities in a dynamic work environment and can more actively face changes in the environment, their work attitudes will become more positive (De Hoogh et al., 2004), and their work will be more productive. Research has found that under the role of work adjustment theory, employees and the work environment often reach a state of conformity. Employees will adjust themselves according to the degree of conformity and show different behavior styles (Bradley et al., 2002). Therefore, when faced with a dynamic work environment, employees will be more likely to pay attention to the opportunities and challenges in the environment, re-understand and evaluate their own environment and current situation, and actively take actions to improve and their job crafting. Their ultimate goal is to be able to carry out and complete work more effectively in a dynamic work environment, and strive to improve innovative performance. As such, the following hypothesis is offered.

**H1: Dynamic work environment has a positive impact on employees’ innovative performance.**

### The Mediating Effect of Job Crafting

According to the purpose of this research, this study constructs the theoretical framework of “dynamic work environment-job crafting-innovative performance”. Job crafting is a kind of “contextualized” activity, embedded in the environment, and its behavior itself and its results are affected by the characteristics of the environment (Zhang and Parker, 2019). Different work situations enable different forms of job crafting to be realized or have different degrees of impact (Wrzesniewski and Dutton, 2001). Job crafting is essentially an employee-led job redesign. They change environment to match their work with their abilities and preferences (Tims and Bakker, 2010). Since job crafting is an initiative of employees, it is described as a personalized, bottom-up, and proactive design method compared with the top-down and “one size fits all” work design methods initiated by the organization (Grant and Parker, 2009; Parker, 2014). The motivations for employees’ job crafting are not only including personal subjective factor but also the influence of job characteristics (Wrzesniewski and Dutton, 2001; Tims et al., 2015). For example, individual needs provide internal motivation for job crafting (Bindl et al., 2019). Employees with high work autonomy have more autonomy in role expansion, idea implementation, and problem solving, which makes it easier to make job crafting (Bizzi, 2017). When environmental changes bring beneficial and challenging work requirements to the personal development of employees, employees will actively implement job crafting (Li et al., 2014).

According to the uncertainty reduction theory, when faced with dynamic changes in the work environment, employees will take a series of actions to reduce the uncertainty they face, so they are more likely to respond to challenges and changes by actively changing work tasks. According to the theory of resource preservation, individuals with more resources will obtain resources in the process of interacting with the surrounding environment, and make full use of various opportunities to create resource surpluses to resist other losses. In other words, in the process of job crafting, employees hope to obtain more resources to achieve resource appreciation, that is, performance improvement.

As a series of behaviors independently carried out by employees, job crafting can improve work performance by increasing work input, adaptability and enthusiasm, and can bring out positive results of person-work matching, and achieving a win-win of work meaning and work performance (Leana et al., 2009; Tims et al., 2015). Studies have shown that job crafting will bring many positive effects, such as helping employees find meaning in their jobs (Wrzesniewski et al., 2013), promoting employees’ work engagement (Vogt et al., 2015), and improving person-work matching (Tims et al., 2016), affecting employees’ innovation performance (Tuan, 2018). According to the theory of human-environment matching and related research on innovative behaviors, job crafting changes the work boundary and content according to the independent wishes of employees, increases the level of work resources-requirement balance, and improves their work commitment and satisfaction. These can improve employees’ efficiency. In the process of job crafting, employees will try new methods to complete work tasks, flexibly use various work resources, and enhance their ability to respond to emergencies at work, thereby enhancing their work performance in a dynamic work environment. The increase in work resources, work engagement and satisfaction can stimulate employees’ intrinsic motivation and encourage them to perform innovative behaviors after completing their jobs efficiently (Demerouti et al., 2015). Individuals increase the matching degree of work with their own abilities, interests and preferences through job crafting, which not only promotes them to give full play to their subjective initiative, but also benefits them to better complete work tasks and improve work performance (Tims and Bakker, 2010; Tims et al., 2015). From the perspective of the three aspects of employee innovation behavior, job crafting has changed the content and boundaries of work. Employees have more opportunities to discover new problems and contradictions in new work situations, which can stimulate the generation of new ideas for employees. Job crafting also includes changing the interpersonal relationships at work and adding more interpersonal resources and interactions. These interpersonal resources can provide support and assistance for employees when new ideas are promoted, and promote employees’ innovative activities (Afsar et al., 2019). At the same time, through the employees’ job crafting, team work efficiency may be improved. In the process of applying employees’ new ideas, team collaboration capabilities are enhanced, which is conducive to the implementation of innovative applications. Therefore, the following hypothesis is offered.

**H2: Job crafting plays a mediating role in the influence of dynamic work environment on employees’ innovative performance.**

### The Moderating Role of Voice Behavior

Research shows that with the deepening of social cognition and psychological capital theoretical research, as well as the profound changes in the employment relationship between organizations and employees, the voice behavior generated by the proactive personality will have an impact on job crafting behavior and innovative performance. The research on voice behavior was first proposed by Hirschman in 1970. He believed that voice behavior was an expression of whether employees were satisfied with their work. The higher the satisfaction, the more inclined employees are to implement voice behavior (Hirschman, 1970). Van et al. (2003) defined the act of voice behavior as proposing ideas and opinions related to one’s own work based on a certain cooperative motivation. From the perspective of the specific content of voice behavior, Liang and Farh (2008) divided the voice behavior into promotive and prohibitive voice behavior, and Janssen and Gao (2015) defined the voice behavior as traditional and novel voice behavior. In terms of composition, voice behavior not only covers the promotive behavior that promotes something to happen, but also covers the prohibitive behavior that promotes the disappearance of something.

In a dynamic work environment, voice behavior is a way of interaction between employees and the organization. On the one hand, voice behavior indicates that employees are encountering cognitive conflicts. On the other hand, it means that employees are open and accepting when facing cognitive conflicts (Ou et al., 2018). First of all, previous studies have shown that employees’ voice behavior increase their work cognitive resources, and the balance between their existing work resources and requirements is broken. Therefore, it is possible to produce job crafting behavior, further improve their work status, and make their work resources more consistent with each other in a new balance. Second, some studies have pointed out that employees’ voice behavior can improve the interpersonal relationship among employees and enable employees to maintain a good working mood (Tjosvold and Su, 2007).Moreover, positive interpersonal relationships and work emotions are a social interpersonal resource at work (Tims et al., 2016). This increase in resources will break the balance of previous work requirements and resources, and drive them to implement job crafting. According to the theory of active behavior motivation, positive emotional attitudes can also drive employees to take active behaviors. Therefore, employees’ voice behavior will have an impact on interpersonal relationships, affect employees’ emotional attitudes at work, and provide positive emotional protection for employees’ job crafting behavior that spontaneously expands their work boundaries. Therefore, the following hypotheses are offered.

**H3a: Promotive voice behavior will positively moderate the impact of the dynamic work environment on job crafting. That is, when employees adopt promotive voice behavior, they are more likely to adopt job crafting in a dynamic work environment.**

**H3b: Prohibitive voice behavior will positively moderate the positive impact of the dynamic work environment on job crafting. That is, when employees adopt prohibitive voice behavior, they are more likely to adopt job crafting in a dynamic work environment.**

Hypothesis H2, H3a, and H3b together construct a mediation model with moderating. As employees’ voice behavior can moderate the relationship between dynamic work environment and job crafting, this research further predicts that the impact of dynamic work environment on innovative performance through job crafting will also be moderated by employees’ voice behavior. In the research on individual performance of employees, many studies believe that employees’ voice behavior can significantly improve process fairness and result fairness (Cohencharash and Spector, 2001), thereby helping employees improve their sense of job control and self-efficacy, and have a positive effect on employees’ innovative performance (Whiting et al., 2008). Studies have shown that employees who regularly implement voice behavior will pay more attention to the status of the organization and think more positively, so they will also get a high level of performance. A study by Ng and Feldman (2012) found that employee’s voice behavior has a strong predictive effect on innovation ability and the implementation of innovative thinking. This paper believes that whether it is promotive or prohibitive voice behavior, it can positively moderate the relationship between job crafting in the dynamic work environment and innovative performance. Therefore, the following hypotheses are offered.

**H4a: Employees’ promotive voice behavior will moderate the mediating role of job crafting in the relationship between dynamic work environment and innovative performance. That is, the higher the level of promotive voice, the stronger the mediating role of job crafting.**

**H4b: Employees’ prohibitive voice behavior will moderate the mediating role of job crafting in the relationship between dynamic work environment and innovative performance. That is, the higher the level of prohibitive voice, the stronger the mediating role of job crafting.**

**FIGURE 1:**
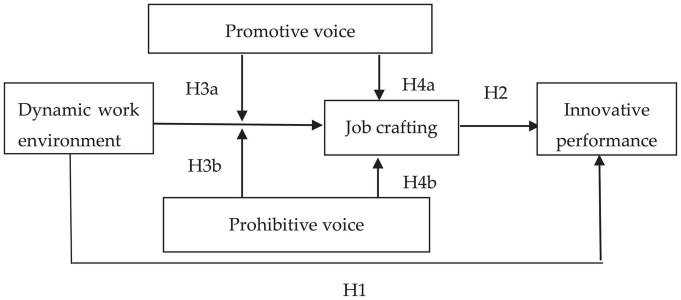
Theoretical model.

In summary, the construction model of this research is shown in Figure 1.

## Research Design

### Research Objects and Questionnaires

The post-epidemic era is a period of normalization and uncertainty. The data collection for this study is in March-April 2021. The research sample mainly selects employees from some companies in the Suzhou National High-tech Industrial Development Zone in China, and has experience of working at home. The formal survey is conducted through a self-managed online questionnaire system and is conducted by random sampling. The formal survey was completed within two months. During the investigation, the respondents were required to answer questions objectively and fairly based on the principle of seeking truth from facts. A total of 240 questionnaires were distributed in this study. After excluding invalid questionnaires, 210 valid questionnaires were obtained, with an effective rate of 87.5%. In terms of sample distribution, the distribution of male and female ratios is relatively balanced. The age distribution is mainly 26-35 years old (53.34% of the total survey population), and the industry and job types are also widely distributed (Table 1).

**TABLE 1:** Demographics of the survey respondents.

Variable		*N*	Percentage	Variable		*N*	Percentage
Gender	Male	108	51.43%	Industry	Electronic information	70	33.33%
	Female	102	48.57%	Industry	New material	35	16.67%
Age	≤ 25	31	14.76%	Industry	Biomedicine	48	22.86%
	[26, 35]	112	53.34%	Industry	New energy	37	17.62%
	≥ 36	67	31.90%	Industry	Others	20	9.52%
Education	High school	50	23.81%	Job	Planning and operation	34	16.19%
	Bachelor degree	125	59.52%	Job	Management	48	22.86%
	Graduate degree	35	16.67%	Job	R&D	51	24.29%
Years	≤ 1	34	16.19%	Job	Finance	32	15.24%
	[2, 5]	90	42.86%	Job	Marketing	45	21.42%
	≥ 6	86	40.95%				

### Scale Design

The scales used in this study are mainly derived from mature scales in the academic community, which have been proven to have good reliability and validity in domestic and foreign studies. All scales use the Likert 5-point scale, 1 means strongly disagree, and 5 means strongly agree. The specific measurement of each variable is as follows.

(1) The independent variable is the dynamic work environment. Using the 3-item scale developed by De Hoogh et al. (2005), the measurement items include “To what extent do you agree that your work environment is very challenging?,” “To what extent do you agree that your work environment is full of changes?” and “To what extent do you agree that your work environment provides many opportunities for change?”. In this study, the Alpha value is 0.893,and the KMO value is 0.876.
(2) The dependent variable is innovative performance. A 10-item scale designed by Janssen (2000), including “I will provide new ideas to improve the current situation,” “I will actively support innovative ideas,” “I can transform innovative ideas into reality application” etc. In this study, the Alpha value is 0.903, and the KMO value is 0.887.
(3) The mediating variable is job crafting. Using Leana et al. (2009) individual job crafting of single-dimensional scale, the scale contains 6 items, such as “I will independently introduce new methods to improve work,” “I will introduce new tasks that I think are more suitable for my skills or interests” etc. In this study, the Alpha value is 0.950, and the KMO value is 0.898.
(4) The moderating variable is voice behavior, including two dimensions: promotive voice behavior and prohibitive voice behavior. Adopting Liang et al. (2012) and Lin and Johnson (2015) who develop a voice behavior scale, including promotive and prohibitive voice items. Among them, the promotive voice behavior has 5 items, such as “I actively put forward a new plan that will benefit the company,” “I actively put forward suggestions to improve the company’s work procedures,” etc. The Alpha value is 0.878, and the KMO value is 0.813. Prohibitive voice behavior has 6 items, such as “I actively report to the leaders the incoordination problems in the work,” “I dare to point out the outdated and inefficient rules and regulations in the company,” etc. The Alpha value is 0.927, and the KMO value is 0.835.

## Data Analysis and Results

### Confirmatory Factor Analysis

In order to ensure that there is a good discrimination validity between the aspects involved in this study, confirmatory factor analysis is used to analyze the competition model on the discrimination effect of dynamic work environment, job crafting, innovative performance, promotive and prohibitive voice behavior. The degree was tested. The results are shown in Table 2. The fitting indicators of the five-factor model are all within the range of the reference standard, and the critical ratio (χ2/df) is the smallest, which is better than other models, indicating that the five constructs involved in this study have good discrimination validity.

**TABLE 2:** Confirmatory factor analysis results.

Construct	Fitness index						
	χ 2/df	GFI	RMSEA	RMR	CFI	NFI	TLI
Five-factor model	2.034	0.959	0.070	0.027	0.979	0.960	0.964
Four-factor model	2.605	0.919	0.056	0.039	0.940	0.906	0.923
Three-factor model	2.905	0.931	0.054	0.048	0.939	0.932	0.930
Two-factor model	3.234	0.826	0.063	0.046	0.875	0.898	0.878
Single-factor model	4.313	0.805	0.071	0.052	0.764	0.785	0.752

### Regression Analysis

This paper uses SPSS to construct a regression model of dynamic work environment and employees’ innovative performance, and conducts regression tests. The results show that the dynamic work environment has a significant positive impact on employees’ innovative performance (β = 0.463, *P* < 0.001). The hypothesis H1 is supported.

### Analysis of the Mediating Effect of Job Crafting

This study used Bootstrap sampling inspection method, set a significance level of 0.05, and sampled 5,000 times to test the mediating effect of job crafting in the impact of dynamic work environment on employees’ innovative performance. The results show that the dynamic work environment can significantly positively affect job crafting (β = 0.305, *P* < 0.001), and job crafting can significantly positively affect innovative performance (β = 0.684, *P* < 0.001). The indirect effect value of dynamic work environment that affects individual innovative performance through job crafting is 0.136, *P* < 0.001, and the 95% confidence interval is [0.180,0.394], excluding 0. The hypothesis H2 is supported.

### Analysis of the Moderating Effect of Voice Behavior

Further, in order to test the moderating effect of voice behavior, the promotive voice behavior, prohibitive voice behavior, and dynamic work environment are first centrally processed, and the interactive items of promotive voice behavior and dynamic work environment are constructed. As well as the interaction terms of prohibitive voice behavior and dynamic work environment are constructed, they are put into the model for testing. The results show that after controlling for the main effects of promotive voice behavior and dynamic work environment, the interaction item of promotive voice behavior and dynamic work environment has a significant positive impact on job crafting (β = 0.490, P < 0.001), indicating hypothesis H3a is supported; After controlling the main effects of prohibitive voice behavior and dynamic work environment, the interaction term of prohibitive voice behavior and dynamic work environment also has a significant positive effect on job crafting (β = 0.316, *P* < 0.001), which shows that the hypothesis H3b is supported.

Finally, a moderated mediation model is constructed with job crafting as the mediating variable and voice behavior as the moderating variable. In this study, the average value of employees’ voice behavior plus or minus one standard deviation was used to select high and low values. Under the high-level of employees’ promotive voice behavior, the indirect effect of the dynamic work environment through job crafting is significant, but under the low-level of employees’ promotive behavior, the indirect effect of the dynamic work environment through job crafting is not significant. From the overall analysis, the difference in indirect effects under the two conditions is significant. So, the hypothesis H4a is supported. In the same way, the hypothesis H4b is also supported. In addition, this paper examines the results of the product of path coefficients. The results of data analysis show that when employees have promotive voice behavior, the indirect effects of dynamic work environment and innovative performance are significant, the moderated mediating effect index is 0.308, and the 95% confidence interval is [0.182, 0.435]. The results of the analysis support that the employees’ promotive voice behavior has a moderating effect on the process of dynamic work environment-job crafting-innovative performance. That is, the higher the employees’ promotive voice behavior, the stronger the mediating role of job crafting in the dynamic work environment and innovation performance. Therefore, the hypothesis H4a is supported. When employees have prohibitive voice behavior, the indirect effects of dynamic work environment and innovative performance are significant, the moderated mediating effect index is 0.292, and the 95% confidence interval is [0.185, 0.410]. The result of the analysis support that employees’ prohibitive voice behaviors has a moderating effect on the process of dynamic work environment-job crafting-innovative performance. That is, the higher the employees’ prohibitive voice behaviors, the stronger the mediating role of job crafting in the dynamic work environment and innovation performance. Therefore, the hypothesis H4b is supported.

## Discussion

This research focuses on the analysis of the mediating mechanism and boundary conditions of the dynamic work environment affecting the employees’ innovative performance. It specifically analyzes the relationship between the dynamic work environment and employees’ innovative performance with job crafting as the mediator and voice behavior as the moderator. Through theoretical research and the empirical research such as SPSS and Amos, the research hypothesis and model construction have been supported. There are also several discussions.

(1) The dynamic work environment has a positive effect on employees’ innovative performance. The dynamic work environment is a realistic situation that employees have to face. In particular, the COVID-19 has made every individual, every organization, and even every country deeply aware of the uncertainty, complexity and dynamics of their environment (Song et al., 2021). In a dynamic work environment, excellent employees are an important factor in promoting the development of an enterprise. The results of this study support the positive effect of a dynamic work environment on employees’ innovative performance. It can be seen that environmental uncertainty is not only a decisive factor that influences organizations on gaining competitive advantage and improving organizational performance, but also brings challenges and opportunities to employees. Employees actively capture opportunities brought by the environment, actively anticipate and respond to challenges, integrate and coordinate job knowledge, and maintain their own competitive advantages (Jacquier-Roux and Paraponaris, 2012). Those employees who can quickly upgrade and adapt to the dynamic environment will be able to fully grasp the current state of the environment, accurately understand the components of the current environment, have a clear judgment on the development trend of innovative activities, and be able to make correct decisions and behaviors. Ultimately, it will improve the innovation performance of employees. Therefore, in the relationship between dynamic work environment and innovative performance, employees should form a good mapping relationship with the dynamic work environment. In a dynamic work environment, employees must not only objectively analyze the challenges and opportunities in innovation activities, but also continuously upgrade themselves to achieve higher performance. Based on this, this research believes that a dynamic work environment will motivate employees to re-examine themselves and the work environment they face, remain vigilant and sensitive to innovation opportunities, proactively respond to environmental challenges, continuously improve their dynamic thinking and strategic decision-making capabilities, which plays a role of instruction and guidance for the improvement of employees’ innovation performance. These findings are consistent with the results of Baron and Tang (2011) and Deng et al. (2019).
(2) Job crafting is an effective way for employees to cope with the dynamic work environment. The results of this study support that job crafting can mediate the positive relationship between dynamic work environment and innovative performance. The results indicate that the impact of dynamic work environment on innovative performance does not occur directly, but requires the mediating effect of job crafting. Logically, this is consistent with the fact that external environmental factors cannot directly affect organizational performance to play a positive role. Just as external environmental factors need to rely on a series of management behaviors of the organization leader to indirectly affect organizational performance. The work environment’s impact on employees is not direct, but requires a series of actions by employees to have an impact on their performance, which is in line with the “environment-behavior-performance” research paradigm. This finding is consistent with the research results of Li and Atuahene-Gima (2001). It can be said that if there is no positive role played by job crafting, even if the dynamic work environment provides employees with potential opportunities, employees will not be able to make effective use of them, and therefore, it will not improve their innovative performance. Based on this, this research believes that between the dynamic work environment and innovation performance, job crafting acts like an “engine” and a “bridge,” playing a transformation and driving role, and is an effective way for employees to cope with the dynamic work environment. At the same time, it also shows that the dynamic work environment has contextualized characteristics of the influence mechanism of employees’ innovation performance. This empirical result also responds to the current debate that “environment-performance” is not a simple linear relationship (Lumpkin and Dess, 2001), and is an affirmative answer to the current research argument that “environmental impact on performance is a complex process.”
(3) Voice behavior is the internal motivation for employees to actively respond to changes. The results of this study show that employees’ promotive and prohibitive voice behaviors both play a positive role in moderating the relationship between the dynamic work environment and job crafting. Subsequently, the research results further support the moderated mediation model. The interaction between the dynamic work environment and the employees’ promotive voice behavior has an impact on employees’ innovative performance through the mediating effect of job crafting. The interaction between the dynamic work environment and employees’ prohibitive voice behaviors also affects employees’ innovative performance through the mediating effect of job crafting. Based on this, this study believes that voice behavior is the internal motivation for employees to take active countermeasures when faced with a dynamic work environment. Whether it is promotive or prohibitive voice behavior, it can play a strengthening role.

## Implications

The results of this study provide important implications.

(1) Both organizations and individuals should actively adapt to the dynamic work environment and inspire their own vitality. It is absolutely not a bad thing that organizations and individuals should pay attention to the dynamic work environment. On the contrary, it may activate the organization and individuals, and prompt individuals to actively make changes to improve innovative performance. Especially when employees are facing greater work pressure, they are in the purpose of protecting their own resources, and they will put forward suggestions that are beneficial to the work, showing more behaviors of seeking resources and less behaviors of reducing requirements. Therefore, when faced with a dynamic work environment, organizations should guide employees to objectively analyze and predict the dynamics and complexity of the work environment. First, the organization must allow employees to fully perceive opportunities and provide them with adequate organizational support to help them tap and use the challenges and opportunities in the environment. Second, organizations should encourage employees to grow and develop independently, give them more space for autonomy, make employees feel self-satisfied and respected, and make work and personal interests more consistent. In this way, employees will show more challenging behaviors and proactive personality, so as to be more proactive in “self-upgrading” to achieve the improvement of innovation performance. In addition, organizations and individuals need to take a long-term view and enhance their environmental sensitivity and adaptability. Although the cultivation and maintenance of these capabilities may require a certain cost, compared with their potential performance, the benefits far exceed the cost.
(2) Organizations must fully understand the importance of job crafting and encourage employees to take the initiative in job crafting. One of the ways to effectively deal with the dynamic work environment is to craft job. Therefore, organizations should make full use of the situation, employees, and job characteristics to allow employees to perceive more opportunities for job crafting, actively adjust work content and relationship boundaries, actively seek resources and challenging work, and reduce inhibitory work requirements. Organizations should give employees appropriate work autonomy and decision-making freedom to motivate employees to implement job crafting, so as to better complete their work tasks. In the work arrangement and design, the organization should enhance the matching of people-work, improve work input and efficiency, and provide employees with resources and substantive assistance, reduce the obstacles they encounter in the process of job crafting, and stimulate innovative behavior. At the same time, organizations must actively meet the needs of employees for job crafting and interpersonal communication, cultivate and train employees’ proactive personality and environmental adaptability, encourage them to actively learn and adapt to the dynamics and complexities faced in work, and improve their response to changes and the ability to solve problems. Organizations also need to actively create a good work environment, such as environmental pressure, leadership style and methods, and organizational policies, to help employees establish organizational identity and role recognition as soon as possible, so as to better engage in work and improve innovation performance. In addition, the organization should provide timely feedback on the behavior and results of employees’ job crafting, and actively encourage, recognize and guide them to form a virtuous circle.
(3) Organizations should improve the management system and mechanism for employees’ voice behavior, and establish a smooth communication channel for employees’ voice behavior. Firstly, it is necessary to establish an organizational mechanism for employees to participate in decision-making, broaden the channels for voice behavior, plan the path of voice behavior, and improve the voice behavior process, so as to ensure the normalization and implementation of voice behavior to a greater extent. Secondly, organizations must reform their organizational structure, strive to implement flat management, ensure the effectiveness, timeliness and smoothness of participation in the system. They must also give full tolerance and respect to voice behaviors. Managers are required to give employees feedback in a timely manner, and adopt reasonable and workable voice. Thirdly, it is necessary to create an open, tolerant and innovative cultural atmosphere, and increase the enthusiasm of employees to make voice behavior. Managers should strive to cultivate non-employment relationships with employees. The basis of employees’ voice behavior is the mutual trust and effective communication between managers and employees, which reduces the psychological barriers for employees to participate in voice behavior. With this, employees can actively put forward new ideas and innovative suggestions. In addition, organizations can consider including the voice behavior into employee performance appraisal indicators, and develop a positive and effective incentive system of voice behavior, so as to encourage employees to continuously improve the content and the quality of their voice behavior, and put forward more targeted voice with commercial value.

## Conclusion

Studies show that the dynamic work environment has a significant positive impact on employees’ innovative performance, and job crafting plays a mediating role in the relationship between the two. In addition, voice behavior positively moderate the relationship between dynamic work environment and job crafting, and the indirect relationship between dynamic work environment and innovative performance through job crafting.

This research still has certain limitations. Future research can be considered from the following aspects. First of all, this study only carried out research based on the research paradigm of “environment-behavior-performance”. It did not consider the impact of the dynamic work environment on employees’ other work attitudes and behaviors, nor did it consider other possible influence mechanisms. Future research can try to explore the different impacts of the dynamic work environment and its impact mechanism, so as to further expand and enrich the research on the impact of the dynamic work environment on employees’ work attitudes and behaviors. For example, studies have shown that in a dynamic work environment, organizational identity, work commitment, feedback seeking behavior, etc., have an impact on employees’ work attitudes and behaviors, which in turn will affect employees’ innovation performance. Second, this study considers the moderating effect of employees’ voice behavior, but this is only one of the boundary conditions that may affect whether employees will actively their job crafting in the face of a dynamic work environment. In fact, there are more factors that will affect the process of action. For example, supervisor support, leadership-member exchange relationships, and employee uncertainty tolerance, etc., may all have an impact on the relationship between the dynamic work environment and innovative performance. Future research can continue to explore the boundary conditions of the dynamic work environment affecting the specific process of innovative performance. For example, with the normalization of the epidemic, there may be new changes in the psychological mood and behavior of employees in the post-epidemic era. At the same time, with the increase in work goals and work complexity, it may be more difficult for employees to manage their own negative emotions. At this time, employees need more organizational context power to provide them with emotional assistance, so as to enhance the positive effects of situational modification and cognitive change. Therefore, future research can focus on the influence mechanism of variables such as organizational context.

## Data Availability Statement

The original contributions presented in the study are included in the article/supplementary material, further inquiries can be directed to the corresponding author/s.

## Ethics Statement

The studies involving human participants were reviewed and approved by Ethics Committee of Wuhan University of Science and Technology. The patients/participants provided their written informed consent to participate in this study.

## Author Contributions

JW distributed the work done in the project, searched the background materials, designed the analytical characterization, and empirical study frame and has done the critical revision and editing.

## Conflict of Interest

The author declares that the research was conducted in the absence of any commercial or financial relationships that could be construed as a potential conflict of interest.

## Publisher’s Note

All claims expressed in this article are solely those of the authors and do not necessarily represent those of their affiliated organizations, or those of the publisher, the editors and the reviewers. Any product that may be evaluated in this article, or claim that may be made by its manufacturer, is not guaranteed or endorsed by the publisher.
